# A six-gene expression signature related to angiolymphatic invasion is associated with poor survival in laryngeal squamous cell carcinoma

**DOI:** 10.1007/s00405-020-06214-1

**Published:** 2020-07-20

**Authors:** Karl Metzger, Julius Moratin, Kolja Freier, Jürgen Hoffmann, Karim Zaoui, Michaela Plath, Fabian Stögbauer, Christian Freudlsperger, Jochen Hess, Dominik Horn

**Affiliations:** 1 Department of Oral and Cranio-Maxillofacial Surgery, Heidelberg University Hospital, Im Neuenheimer Feld 400, 69120 Heidelberg, Germany; 2 Department of Oral and Cranio-Maxillofacial Surgery, Saarland University Hospital, Kirrberger Str. 100, 66421 Homburg, Germany; 3 Department of Otorhinolaryngology, Heidelberg University Hospital, Im Neuenheimer Feld 400, 69120 Heidelberg, Germany; 4 Institute of Pathology, Technical University of Munich, TUM School of Medicine, Trogerstr. 18, 81675 Munich, Germany; 5 Department of Otorhinolaryngology, Section Experimental and Translational Head and Neck Oncology, Research Group Molecular Mechanisms of Head and Neck Tumors, German Cancer Research Center (DKFZ), Heidelberg University Hospital, Im Neuenheimer Feld 400, 69120 Heidelberg, Germany

**Keywords:** Head and neck squamous cell carcinoma, Head and neck cancer, Angiolymphatic invasion, Laryngeal squamous cell carcinoma, HNSCC

## Abstract

**Purpose:**

Angiolymphatic invasion serves as a histopathological risk factor for unfavorable survival in head and neck squamous cell carinoma. The aim of the study was to explore  the molecular mechanisms characterizing angiolymphatic invasion and therefore identify a gene expression signature related to angiolymphatic invasion.

**Methods:**

Gene expression analysis of head and neck squamous cell carcinoma was carried out based on clinical and whole genome expression data provided by The Cancer Genome Atlas. Results were validated in an independent cohort of laryngeal squamous cell carcinoma and confirmed by immunohistochemistry staining.

**Results:**

A gene expression signature consisting of six genes (*SHH, SLC18A3, LCE3E, LCE2B, LCE3D* and *DSG-1*) related to angiolymphatic invasion was identified. The gene expression profile identified a subset of patients with decreased overall survival (*p* = 0.02, log rank test), which was most prominent for patients with laryngeal squamous cell carcinoma (*p* = 0.004, log rank test). Furthermore, these patients showed a significant shorter progression-free survival (*p* = 0.002, log rank test). By use of this gene expression signature, patients at high risk of recurrence could be identified even if morphological changes were not yet recognizable.

**Conclusion:**

Angiolymphatic invasion is characterized by a distinct histopathological phenotype and specific gene expression signature. The newly identified signature might serve as a reliable predictor of outcome in laryngeal cancer and add additional benefit to histopathological evaluation.

**Electronic supplementary material:**

The online version of this article (10.1007/s00405-020-06214-1) contains supplementary material, which is available to authorized users.

## Introduction

Head and neck squamous cell carcinomas (HNSCC) are affecting more than 600,000 patients per year [1, 2]. HNSCC arises from the mucosal lining of the upper aerodigestive tract, including the oral cavity (OSCC), oropharynx (OPSCC) and larynx (LaSCC). Tobacco smoking, alcohol consumption, betel nut chewing and human papillomavirus (HPV) infection are the main risk factors for the development of HNSCC [3–6]. As a result of multi-modal therapy, 5-year survival rates have increased up to 67% for all stages during the last decades [7, 8]. However, survival rates of laryngeal cancer decreased over the past decades from 66 to 63% [9], with an unfavorable prognosis for advanced stages [10, 11]. This is important, as most patients suffering from laryngeal carcinoma present with advanced disease (stage III/IV) at the time of diagnosis [12]. This emphasizes the need for innovations in the field of risk stratification with subsequent therapy adjustments.

Surgical therapy plays a fundamental role in the treatment of HNSCC and is complemented by radiation and chemotherapy in advanced cases (stage III/IV) [8]. Relevant prognostic factors in HNSCC are clinical and histological characteristics, such as tumor size and localization, lymph node extracapsular spread (ECS), and perineural (PNI) and angiolymphatic invasion (ALI) [13–16]. Two of these histological characteristics have been investigated at a molecular level. An 11 gene expression profile for extracapsular spread in oral squamous cell carcinoma serves as a prognosticator of outcome in patients without nodal metastases [17]. In terms of PNI, a gene expression profile revealed a subset of HNSCC at risk of post-surgical recurrence [18]. However, little is known about the molecular mechanisms related to ALI and a molecular signature for ALI has not been identified so far. ALI implies tumorous invasion of lymphatic vessels and intratumoral vascular invasion. It is correlated with higher rates of lymph node metastases and poor prognosis in oral squamous cell carcinoma [19].

The main objective of this study was to explore the molecular mechanisms of ALI and to identify a gene expression signature associated with ALI. Although ALI is a common histopathological characteristic and predictor of outcome in HNSCC, histopathological examination implies a level of uncertainty. It depends on a single pathological examination, and extensive specimens are needed for robust classification. Especially, small biopsy specimens do not meet these demands.

Therefore, a molecular indicator for patients at high risk of recurrence may support histopathological examination, alter therapeutic strategies and improve patient’s prognosis. We investigated transcriptome data of HNSCC in relation to the occurrence of ALI and patient’s survival based on a global gene expression analysis using TCGA data. We also confirmed newly identified candidate genes in an independent cohort of LaSCC. The whole genome expression data of this cohort were collected in collaboration with the German cancer research center and the Heidelberg Center for Personalized Oncology (DKFZ-HIPO).

## Material and methods

### Data acquisition

#### TCGA cohort

A publicly available dataset containing clinical and pathological information on patients with HNSCC was published by the TCGA Research Network. All patients were treated by surgery, followed by radiotherapy or radio-chemotherapy if needed. mRNA count data containing 19,750 genes of 500 patients with HNSCC as well as general clinical and pathological characteristics were downloaded from https://portal.gdc.cancer.gov on January 25, 2019. Updated survival data were retrieved from https://gdc.cancer.gov/about-data/publications/pancanatlas on March 14, 2019. Patients with missing subsite information were excluded from further analysis, resulting in a total of 497 patients.

#### HIPO-HNC cohort

Patients with histological confirmed LaSCC (*n* = 13) of the HIPO-HNC cohort were treated at the Heidelberg University Hospital between 2012 and 2016. The seldom expression data of LaSCC were collected in collaboration with the German cancer research center and the Heidelberg Center for Personalized Oncology (DKFZ-HIPO). Patient samples were obtained under the protocol S-206/2011, approved by the Ethics Committee of Heidelberg University, with written informed consent from all participants. This study was conducted in accordance with the Declaration of Helsinki. Sample processing and generation of array-based expression data have been described previously [20]. These data have been uploaded to NCBI's Gene Expression Omnibus and are accessible through GEO Series accession number GSE117973. The clinical and pathological information are summarized in Supplemental table S1. Formalin-fixed paraffin-embedded tissue specimens were retrieved from the tissue bank of the National Center for Tumor Diseases, Heidelberg, Germany.

### Data analysis

#### Differential gene expression (DGE)

The DGE analysis was performed on the TCGA cohort in R version 3.5.1 using the packages DESeq2 and limma [21, 22]. Genes with an FDR less than 0.05 and a log2-fold-change greater 1.5 were considered as significantly differentially expressed. FDR values were adjusted using Benjamini–Hochberg correction. The results of DESeq2 and limma were compared, and consistent genes were used for further analysis.

#### Unsupervised hierarchical clustering

Transcript data of selected genes were used for unsupervised hierarchical clustering and to construct a heatmap via ClustVis, a web tool for visualizing multivariate data. Hierarchical clustering was performed using Pearson correlation [23].

#### Chi-squared test and univariate and multivariate analysis

Chi-squared test was used for comparisons of categorical data and a *p* value < 0.05 was considered as statistically significant. Univariate and multivariate analysis was carried out using the Cox regression model. All analyses were performed in R version 3.5.1.

#### Survival analysis

Overall and progression-free survival was estimated using the Kaplan–Meier method. Log-rank test was used to compare the differences between groups. *p* values less than 0.05 were considered statistically significant. Overall survival was defined as a time period from the date of diagnosis to the last date when the patient was known to be alive (censored) or date of death for any reason (uncensored). Progression-free survival was measured from the time of diagnosis to the day of the last follow‐up examination in which the patient was progression free (censored), or to the date of local recurrence of the disease or occurrence of regional or distant metastases (uncensored). Survival analysis was performed in R version 3.5.1 using the packages survminer, survival and ggplot2.

### Immunohistochemistry

Immunohistochemical staining was carried out on 4 μm-thick sections from FFPE tissue specimens of the HIPO-HNC cohort. IHC staining was done as described previously [24]. GLI2 was detected by mouse monoclonal antibodies (Santa Cruz Biotechnology, Dallas, USA, clone sc-271786, 1:100 in PBS) and DSG1 was detected by mouse monoclonal antibody (Progen, clone 18107; 1:10 in PBS). The analysis was carried out by two independent observers.

## Results

### Association between ALI and progression free or overall survival

**Fig. 1:**
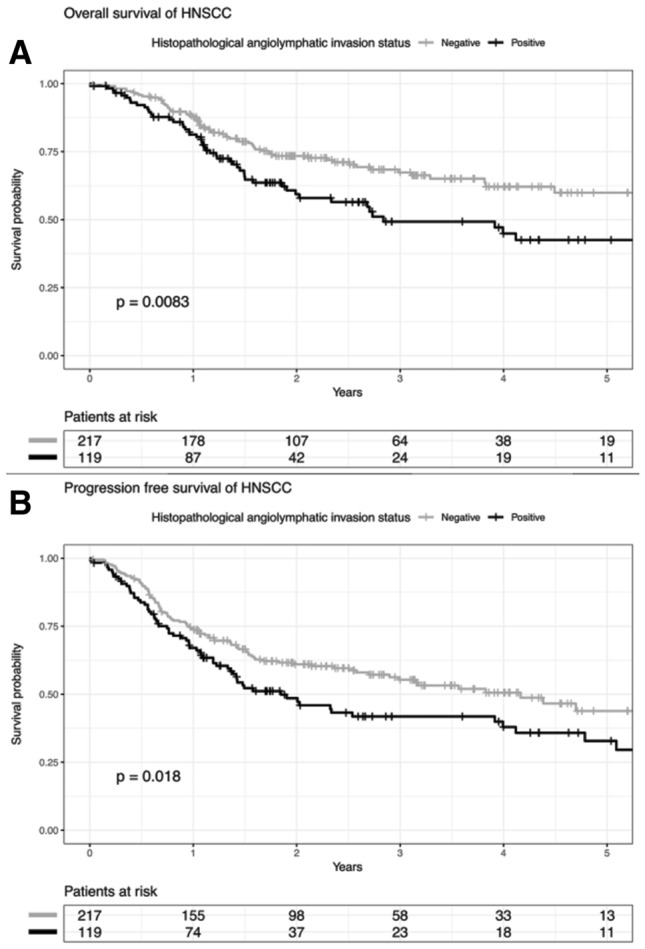
**a** Overall survival of patients in the TCGA-HNSCC cohort according to angiolymphatic invasion status. **b** Progression-free survival of patients in the TCGA-HNSCC cohort according to angiolymphatic invasion status

A histological ALI status was available for 336 out of 497 (68%) patients of the TCGA-HNSC cohort. The impact of ALI on survival was assessed by Kaplan–Meier analysis and log rank test to confirm a statistically significant shorter progression-free (*p* = 0.018, log rank test) and overall survival (*p* = 0.008, log rank test) for cases with ALI as compared to ALI-negative counterparts (Fig. 1a, b). The subgroup analysis demonstrated that the association between ALI and recurrent disease or overall survival was evident for LaSCC (PFS: *p* = 0.047, OS: *p* = 0.036, log rank test) and OSCC (PFS: *p* = 0.021, OS: *p* = 0.02, log rank test), but not for OPSCC (PFS: *p* = 0.98, OS: *p* = 0.61, log rank test) (Suppl. Fig. S1A–F).

### ALI-related gene expression signature

A differential gene expression analysis was performed for subgroups with positive and negative histopathological ALI status (*n* = 336) and significant candidate genes (FDR < 0.05 and − 1.5 < log2-fold-change > 1.5) were considered for further analysis. 93 genes were identified by the DESeq2 package (60 with increased mRNA expression level, 33 with decreased mRNA expression level), and 17 genes were identified by the limma package (two with increased mRNA expression level, 15 with decreased mRNA expression level). To reduce the number of genes and to identify robust candidates, consistent candidate genes were selected. In total, six differentially expressed genes (*SHH* and *SLC18A3* with increased mRNA expression level; *LCE3E, LCE2B, LCE3D* and *DSG-1* with decreased mRNA expression level) were identified by both packages and selected for further analysis (Suppl. tables S2–3).

We hypothesized that the transcript levels of the six candidate genes are sufficient to predict the risk of ALI in all patients of the TCGA cohort (*n* = 497). Indeed, unsupervised hierarchical clustering using Pearson correlation revealed two main clusters (Suppl. Fig. S2). While tumors in cluster A (264 out of 497; 53%) were enriched for a negative ALI status, most tumors in cluster B (233 out of 497; 47%) had a positive ALI status. These data demonstrated the potential of the six-gene expression signature to stratify tumors reliably by ALI status (*p* = 2.451e−09, Chi-squared test, Suppl. tables 4–5). Furthermore, previously unclassified patients (*n* = 161) could be categorized to either cluster A or cluster B by use of the presented gene signature. Therefore, the six-gene expression signature enabled segregation of cases for which histological information on the ALI status was not available.

### ALI-related gene expression signature predicts tumor recurrence and unfavorable survival in laryngeal carcinoma

**Fig. 2:**
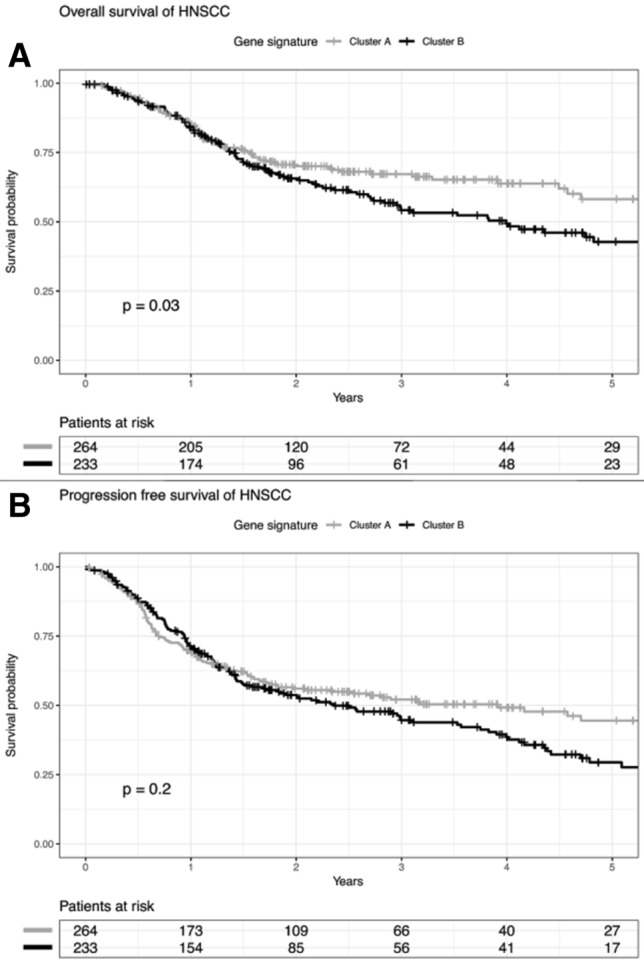
**a** Overall survival of patients in the TCGA-HNSCC cohort according to gene expression signature. **b** Progression-free survival of patients in the TCGA-HNSCC cohort according to gene expression signature

**Fig. 3:**
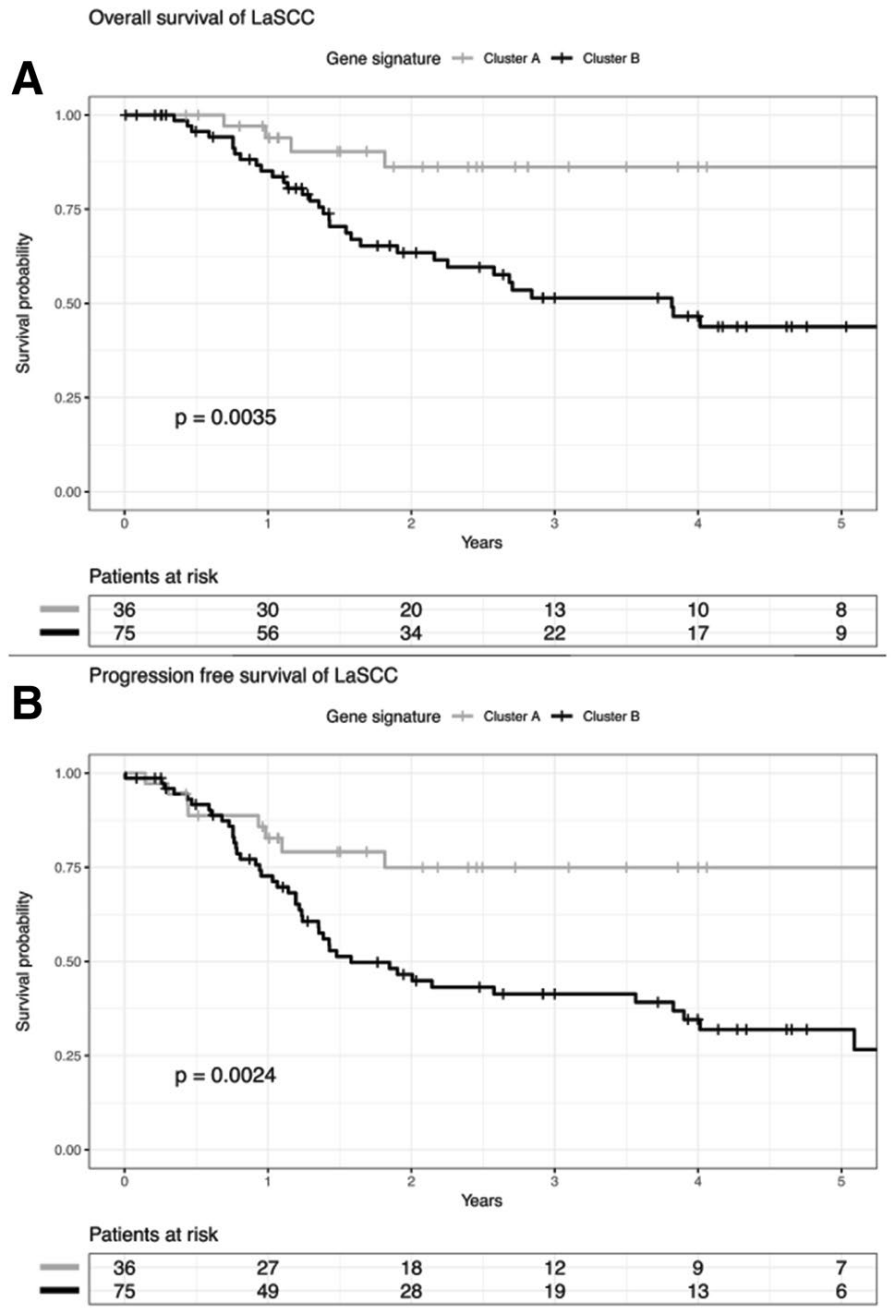
**a** Overall survival of patients in the TCGA-LaSCC cohort according to gene expression signature. **b** Progression-free survival of patients in the TCGA-LaSCC cohort according to gene expression signature

**Fig. 4:**
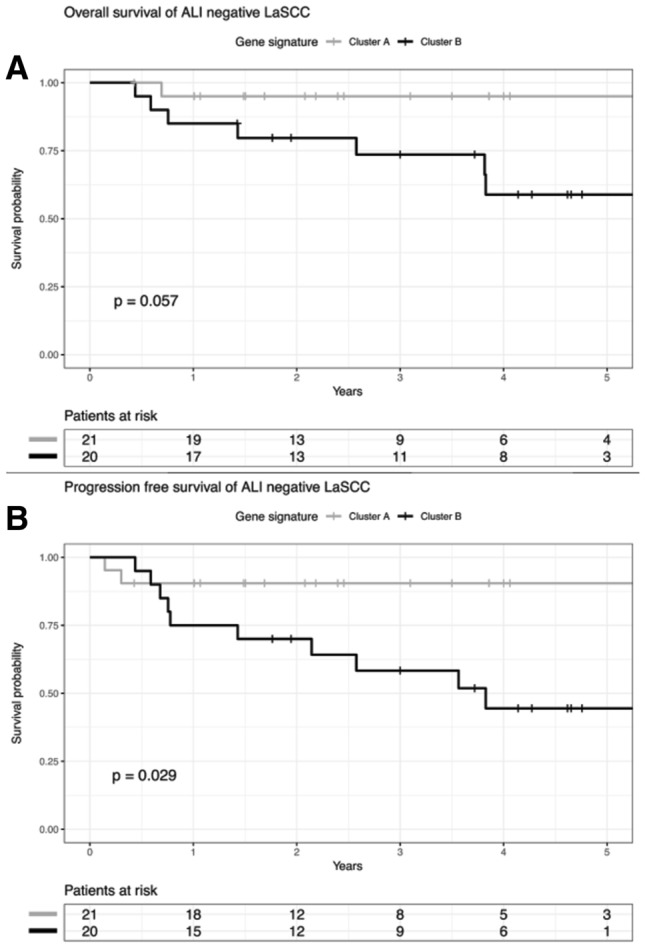
**a** Overall survival of patients with confirmed negative histopathological ALI status in the TCGA-LaSCC cohort according to gene expression signature. **b** Progression-free survival of patients with confirmed negative histopathological ALI status in the TCGA-LaSCC cohort according to gene expression signature

As the six-gene signature stratifies HNSCC reliably according to the ALI status, we addressed the question whether patients in clusters A and B differ in prognosis. All patients of the TCGA-HNSCC (*n* = 497) cohort were considered, including both patients with and without available information on the histological ALI status. Kaplan–Meier plots revealed a significant shorter overall survival for patients in cluster B (*p* = 0.02, log rank test) (Fig. 2a, b; Supplemental tables 6–7). The subgroup analysis considering distinct subsites of HNSCC demonstrated that differences in overall survival were most prominent for patients with LaSCC (*p* = 0.004, log rank test). Moreover, LaSCC patients in cluster B shared a significant shorter progression-free survival (*p* = 0.002, log rank test) (Fig. 3a, b, Supplemental table 8–9). Interestingly, 20 out of 41 (49%) LaSCC patients with a negative ALI status as determined by histological inspection were part of cluster B according to the six-gene expression signature. These patients showed unfavorable PFS (Fig. 4a, b), indicating a substantial amount of patients at risk with a lacking histomorphologic ALI-like phenotype. In contrast, only 4 out of 34 (12%) LaSCC patients with a histologically positive ALI status were part of cluster A according to the six-gene signature (Supplemental table 10).

In conclusion, the newly identified expression signature consisting of six genes is closely associated with ALI and stratifies HNSCC patients at a higher risk for unfavorable progression-free and overall survival, in particular for the subgroup of LaSCC patients, even in histologically unclassified patients.

### Univariate and multivariate analysis of laryngeal subsite

Univariate analysis and multivariate Cox regression models were conducted to identify independent risk factors regarding overall and progression-free survival in patients with LaSCC (Table 1). Cluster B, females, positive resection margins, extracapsular spread and perineural invasion were parameters significantly associated with unfavorable OS and PFS in the univariate analysis. In addition, a positive ALI status and non-smoking status were significantly associated with poor OS. ALI status was excluded from the multivariate analysis as it is not independent from the presented gene expression signature. Extracapsular spread and perineural invasion were excluded from the multivariate analysis due to missing information in a large proportion of patients. Non-smoking status (HR 0.11, *p* < 0.001), females (HR 3.47, *p* = 0.010) and positive resection margins (HR 5.64, *p* < 0.001) were associated with poor OS in multivariate analysis. Cluster B (HR 2.5, *p* = 0.016) and positive resection margins (HR 2.46, *p* = 0.026) were the only parameters associated with unfavorable PFS in multivariate analysis.

**Table 1:** Univariate and multivariate analysis of overall and progression-free survival of LaSCC (*n* = 111)

Variable	Univariate analysis			Multivariate analysis		
	HR	CI	*p* value	HR	CI	*p* value
*OS*						
Tobacco yes vs. no	**0.2242**	**0.0752–0.6685**	**0.00731**	**0.1123**	**0.03323–0.3791**	**0.000429**
Alcohol	0.8485	0.4516**–**1.594	0.61			
Cluster B vs. A	3.168	1.403**–**7.155	0.00552	2.3603	0.94778**–**5.8778	0.065073
Age	1.017	0.9794**–**1.057	0.376			
Gender f vs m	**3.211**	**1.54–6.696**	**0.00187**	**3.4690**	**1.33880–8.9885**	**0.010449**
R1 vs R0	**4.579**	**1.998–10.49**	**0.000324**	**5.6384**	**2.2861–13.9066**	**0.000173**
Grade	1.004	0.6897**–**1.462	0.983			
ALI + vs −	2.279	1.031**–**5.039	0.0418			
ECS + vs −	2.454	1.147**–**5.248	0.0207			
PNI + vs −	3.78	1.568**–**9.108	0.00305			
Stage III/IV vs I/II	0.7174	0.2476**–**2.079	0.541			
*PFS*						
Tobacco yes vs. no	0.5149	0.1823**–**1.455	0.21			
Alcohol yes vs. no	0.8569	0.498**–**1.474	0.577			
Cluster B vs. A	**2.795**	**1.4–5.582**	**0.00357**	**2.499**	**1.1874–5.261**	**0.0159**
Age	0.9913	0.961**–**1.023	0.582			
Gender f vs m	2.252	1.172**–**4.326	0.0148	2.107	0.9592**–**4.627	0.0634
R1 vs R0	**2.91**	**1.325–6.392**	**0.00781**	**2.455**	**1.1113–5.423**	**0.0264**
Grade	1.015	0.7184**–**1.435	0.931			
ALI	2.063	0.9957**–**4.275	0.0514			
ECS + vs −	2.397	1.141**–**5.036	0.021			
PNI + vs −	3.118	1.446**–**6.722	0.00372			
Stage III/IV vs I/II	1.016	0.3974**–**2.596	0.974			

Variables identified as independent prognostic factors by multivariate analysis are highlighted

*HR* hazard ratio, *CI* 95% confidence interval

### Validation in an independent LaSCC cohort

The independent validation cohort consisted of 13 patients with LaSCC, who were treated between 2012 and 2016 at the University Hospital Heidelberg (HIPO-HNC). Eleven patients (85%) were male and two patients were female (15%). Patients’ age ranged from 49 to 86 years with a median age of 61 years. All patients had a positive smoking history. 6 out of 13 (46%) patients reported alcohol consumption. Histopathological ALI status was positive in 3 out of 13 (23%) patients (Supplemental table 11). For all tumors, global gene expression data were available and used to conduct unsupervised hierarchical clustering based on the six-gene expression signature. Again, the six-gene expression signature revealed two clusters, and all cases with a positive ALI status based on histological examination were part of cluster B (Suppl. Figure 3). Four out of ten patients with a histologically negative ALI status were part of cluster B, indicating specimens lacking histomorphological ALI-like phenotype. However, due to the low number of patients, no survival analysis was conducted for the HIPO-HNC cohort.

### Immunohistochemical detection of GLI2 and DSG1

To confirm altered expression of selected candidate genes of the six-gene expression signature on protein level, GLI2 and DSG-1 protein expression was detected by immunohistochemical staining on tumor sections of the HIPO-LaSCC cohort. GLI2 is well established as a downstream target of SHH and was used as a surrogate marker for active SHH signaling. IHC staining confirmed high GLI2, but low DSG1 protein expression in cancer cells of LaSCC attributed to cluster B and an opposite staining pattern for cases in cluster A (Suppl. Figure 4).

In conclusion, the newly identified gene expression signature is associated with histological ALI status in patients with HNSCC and segregates both patients with and without available histological ALI status. It serves as a prognosticator of poor OS in HNSCC and predicts short progression-free and overall survival in LaSCC. In addition, patients at risk with lacking ALI-like phenotype could be identified. Findings could be confirmed in an independent cohort of LaSCC.

## Discussion

The purpose of this study was to identify a specific gene expression signature associated with ALI in HNSCC. Therefore, we analyzed publicly available data of the TCGA-HNSCC cohort. Microscopically determined ALI was associated with a significant shorter progression-free and overall survival in the investigated cohort. This is consistent with current evidence as ALI is a known prognosticator of outcome and patients with ALI-positive HNSCC are recommended to undergo adjuvant radiotherapy [25, 26]. The conducted gene expression analysis unveiled a six-gene expression signature, which was closely related to ALI status and predicted tumor recurrence as well as unfavorable survival in HNSCC, in particular for the subsite of LaSCC. Previous studies reported gene expression signatures in association with perineural invasion and extracapsular spread in different subpopulations of HNSCC [17, 18]. To our knowledge, this is the first study on an ALI-related gene expression signature in the field of HNSCC.

With regard to clinical relevance and in line with the close association to ALI, the newly identified six-gene expression signature served as a risk factor for unfavorable prognosis. Out of the TCGA-HNSCC cohort, 233 patients (47%) could be identified at a significantly higher risk of mortality. Moreover, the application of this gene signature on subsites of HNSCC revealed patients with LaSCC at a high risk of recurrence and poor overall survival. It is important to emphasize that patients showed recurrence in the presence of this gene signature even in ALI-negative tumors, according to pathological assessment. In addition, patients lacking histological ALI status were classified to either cluster A or cluster B by the presented gene signature. This underlines the additional benefit of the presented signature, as some specimens lack an ALI-like phenotype, while patients experience unfavorable survival.

Pathological examination includes examination of extensive and heterogeneous specimens in patients treated by tumor resection. Traditional risk stratification of patients treated by primary radio(chemo)therapy is challenging, as only small biopsy specimens are available for pathological examination. By use of the presented six-gene signature, biopsy specimen could be analyzed for further characterization of this subgroup of patients. In addition, molecular profiling of circulating tumor cells may be useful in the course of liquid biopsies in the future.

The identified six-gene expression signature includes two genes (SHH, SLC18A3) with increased mRNA expression levels and four genes (LCE3E, LCE2B, LCE3D, DSG1) with decreased mRNA expression levels, suggesting a gain in SHH signaling accompanied by a loss of epithelial differentiation is one underlying principle of ALI. The activation of hedgehog signaling has been described as a prognostic indicator in oral squamous cell carcinoma [27]. An increase in SHH signaling results in cellular proliferation and transformation and it has been described as an indirect angiogenic factor [28, 29]. Angiogenesis as a consequence of SHH function results in robust neovascularization by the induction of an array of angiogenic growth factors, including all isoforms of VEGF [28]. High lymphatic vessel density is correlated with poor OS and lymph node metastasis in HNSCC [30, 31].

SLC18A3 encodes a vesicular acetylcholine transporter (VAChT). The presence of VAChT in epidermal melanocytes and keratinocytes has been demonstrated [32]. Vesamicol, an VAChT antagonist, induces potent apoptosis in bronchioalveolar carcinoma [33]. As cholinergic signaling is upregulated in SCC and blocking cholinergic signaling limits growth of squamous cell lung carcinoma, cholinergic signaling is a potential target in the treatment of HNSCC [34].

All genes in the signature with an inverse correlation to ALI are linked to epithelial differentiation. As a member of desmosomal cadherins, desmoglein-1 (DSG1) is important in epithelial differentiation and intercellular junction mediating a strong cell–cell adhesion [35]. A loss of DSG1 is associated with an invasive phenotype, metastasis and decreased survival in squamous cell carcinoma [36–40].

We confirmed high GLI2, but low DSG1 protein expression in tumors of cluster B by immunohistochemical staining, which was enriched for ALI-positive cases. The presence of a different expression pattern on protein level could support standard histological examination to determine the risk for ALI.

Our results have some limitations. We relied on publicly available data which are subject to multiple errors, including data collection in multiple centers, pathologic examination by different pathologists and recall bias. We are aware of a limited number of patients in our in-house cohort. However, we could not identify a larger cohort of LaSCC containing all six genes of the described gene signature. Moreover, the availability of tissue specimen is a huge advantage of this cohort. While we showed that both low expression levels of genes affecting epidermal differentiation and high expression level of SHH are related to ALI, further studies may clarify whether SHH affects transcription of these epidermal markers.

The presented gene expression signature identifies histological ALI-negative patients at risk of disease progression and poor overall survival. This gene expression signature may serve as a molecular surrogate for ALI, as it reliably stratifies patients by ALI status. Furthermore, it enables risk stratification of patients lacking ALI status and predicts outcome in cases with missing ALI-like phenotype. Hereby, adjuvant therapy might be escalated or de-escalated according to the presented signature.

## Conclusion

We identified a novel gene expression signature related to ALI status in HNSCC patients. This gene expression signature identifies HNSCC patients at risk of poor overall survival and predicts tumor recurrence and unfavorable survival in laryngeal carcinoma, even if histological ALI status was negative or unknown. However, as our independent validation cohort is small, further validation in a large prospective cohort is a major objective for further studies. In the future, implementation of additional stratification tools could assist in decision making in adjuvant therapy resulting in better prognosis of patients in need.

## Electronic supplementary material

Below is the link to the electronic supplementary material.

Supplemental figure 1. Subgroup analysis of overall and progression-free survival of patients out of the TCGA-HNSCC cohort according to angiolymphatic invasion status. (EPS 5974 kb)

Supplemental figure 2. Heatmap showing the expression levels of the six signature genes in patients out of the TCGA-HNSCC cohort. Two separate main clusters A and B are apparent. (EPS 242 kb)

Supplemental figure 3. Heatmap showing the expression levels of the six signature genes in patients out of the HIPO-LaSCC cohort. Two separate main clusters A and B are apparent. (EPS 18 kb)

Supplemental figure 4. Representative immunohistochemical staining against DSG1 (A+C) and GLI2 (B+D) on Cluster A (A+B) and Cluster B (C+D) tumor sections of the HIPO-LaSCC cohort. (TIF 76084 kb)

Supplementary file5 (DOCX 24 kb)
