# Correction to: A six-gene expression signature related to angiolymphatic invasion is associated with poor survival in laryngeal squamous cell carcinoma

**DOI:** 10.1007/s00405-021-07131-7

**Published:** 2021-10-19

**Authors:** Karl Metzger, Julius Moratin, Kolja Freier, Jürgen Hofmann, Karim Zaoui, Michaela Plath, Fabian Stögbauer, Christian Freudlsperger, Jochen Hess, Dominik Horn

**Affiliations:** 1 Department of Oral and Cranio-Maxillofacial Surgery, Heidelberg University Hospital, Im Neuenheimer Feld 400, 69120 Heidelberg, Germany; 2 Department of Oral and Cranio-Maxillofacial Surgery, Saarland University Hospital, Kirrberger Str. 100, 66421 Homburg, Germany; 3 Department of Otorhinolaryngology, Heidelberg University Hospital, Im Neuenheimer Feld 400, 69120 Heidelberg, Germany; 4 Institute of Pathology, Technical University of Munich, TUM School of Medicine, Trogerstr. 18, 81675 Munich, Germany; 5 Department of Otorhinolaryngology, Section Experimental and Translational Head and Neck Oncology, Research Group Molecular Mechanisms of Head and Neck Tumors, German Cancer Research Center (DKFZ), Heidelberg University Hospital, Im Neuenheimer Feld 400, 69120 Heidelberg, Germany

## Correction to: European Archives of Oto-Rhino-Laryngology (2021) 278:1199–1207 10.1007/s00405-020-06214-1

The article A six-gene expression signature related to angiolymphatic invasion is associated with poor survival in laryngeal squamous cell carcinoma, written by Karl Metzger, Julius Moratin, Kolja Freier, Jürgen Hofmann, Karim Zaoui, Michaela Plath, Fabian Stögbauer, Christian Freudlsperger, Jochen Hess and Dominik Horn, was originally published Online First without Open Access. After publication in volume 278, issue 4, page 1199–1207 the author decided to opt for Open Choice and to make the article an Open Access publication. Therefore, the copyright of the article has been changed to © The Author(s) 2021 and the article is forthwith distributed under the terms of the Creative Commons Attribution 4.0 International License which permits use, sharing, adaptation, distribution and reproduction in any medium or format, as long as you give appropriate credit to the original author(s) and the source, provide a link to the Creative Commons license, and indicate if changes were made. The images or other third party material in this article are included in the article’s Creative Commons licence, unless indicated otherwise in a credit line to the material. If material is not included in the article’s Creative Commons licence and your intended use is not permitted by statutory regulation or exceeds the permitted use, you will need to obtain permission directly from the copyright holder. To view a copy of this licence, visit http://creativecommons.org/licenses/by/4.0. Open access funding enabled and organized by Projekt DEAL.

The original article has been updated.

