# Supplementary material for: A six-gene expression signature related to angiolymphatic invasion is associated with poor survival in laryngeal squamous cell carcinoma
Source: Eur Arch Otorhinolaryngol. 2020 Jul 20;278(4):1199–207. doi: 10.1007/s00405-020-06214-1 (PMC8519817; doi:10.1007/s00405-020-06214-1)
Supplement: Supplementary file 5 — Supplementary file5 (DOCX 24 kb) [file 405_2020_6214_MOESM5_ESM.docx]

**Supplemental tables**

Supplemental table 1: Clinical characteristics of our in-house cohort of LaSCC

| **Characteristics** | **LaSCC** |
| --- | --- |
|  | **Number of patients n=13** |
| Sex  Male  Female | 11 (85%)  2 (15%) |
| Median age (range) | 61 (49-83) |
| Smoking history  Yes  No | 13 (100%)  0 (0%) |
| Alcohol history  Yes  No | 6 (46%)  7 (54%) |
| Tumor size  T1  T2  T3  T4 | 0 (0%)  0 (0%)  6 (46%)  7 (54%) |
| Nodal status  N0  N1  N2  N3  ECS+ | 8 (62%)  1 (8%)  4 (31%)  0 (0%)  3 (23%) |
| UICC  I+II (limited)  III+IV (advanced) | 0 (0%)  13 (100%) |
| Grading  G1  G2  G3  G4 | 0 (0%)  11 (85%)  2 (15%)  0 (0%) |
| Angiolymphatic invasion  Positive  Negative | 3 (23%)  10 (77%) |
| Perineural invasion  Positive  Negative | 0 (0%)  13 (100%) |
| Therapy  Surgery  Surgery + Radiotherapy  Surgery + Radiochemotherapy | 4 (31%)  4 (31%)  5 (38%) |
| Surgical margins  R0  R1 | 12 (92%)  1 (8%) |

Supplemental table 2: Differentially expressed genes of TCGA-HNSCC cohort according to angiolymphatic invasion status using DESeq2 package

| **Hugo Symbol** | **log2 Fold Change** | **FDR** |
| --- | --- | --- |
| SPAG6 | 2,033025548 | 3,35E-14 |
| PIH1D3 | 2,055664332 | 2,58E-07 |
| NEFH | 1,999706014 | 2,62E-12 |
| TAF7L | 1,764736425 | 3,07E-10 |
| MT4 | 1,748566711 | 4,62E-05 |
| TOX3 | 1,669334412 | 4,84E-06 |
| DBX1 | 1,688784033 | 0,00011244 |
| APOC3 | 1,546179048 | 0,00325642 |
| SCGB2A2 | 1,986825852 | 5,90E-06 |
| PRR4 | 2,039172788 | 4,29E-18 |
| GHRH | 1,955693118 | 0,00028672 |
| C20orf85 | 2,434652442 | 1,42E-07 |
| CRISP2 | 2,004871378 | 8,42E-05 |
| SCGB2A1 | 1,937115694 | 1,93E-09 |
| KLK14 | -1,722130827 | 1,06E-09 |
| KIF1A | 2,103076573 | 3,30E-12 |
| DDC | 2,20812573 | 1,76E-16 |
| PRH2 | 3,389210638 | 8,87E-24 |
| DSG1 | -1,525842188 | 3,65E-09 |
| SIX3 | 1,604965055 | 1,86E-08 |
| SPINK7 | -2,024571637 | 3,22E-10 |
| CPLX2 | 1,584418564 | 3,72E-05 |
| GFRA3 | 2,068746406 | 3,25E-17 |
| SLC22A8 | 1,607637557 | 7,78E-06 |
| C1orf158 | 1,734196131 | 9,71E-08 |
| LCE2B | -1,65963775 | 0,00019445 |
| TFF3 | 1,689945542 | 9,87E-11 |
| TFF1 | 1,520125206 | 0,00020177 |
| RSPH1 | 1,512410934 | 3,73E-15 |
| FTCD | 1,51114276 | 3,27E-08 |
| CCDC155 | 2,106819664 | 8,40E-10 |
| ALOX15 | 1,536610867 | 6,93E-08 |
| FGF19 | 2,371459904 | 6,06E-10 |
| LCE3D | -1,605878791 | 1,19E-08 |
| SHH | 2,659760656 | 3,39E-19 |
| GBX1 | 1,521839707 | 0,00139885 |
| ZMAT4 | 1,618042659 | 9,68E-07 |
| CLDN3 | 1,749374646 | 2,64E-08 |
| ARMC3 | 2,110542209 | 1,39E-11 |
| SERPINB12 | -1,7173925 | 3,59E-09 |
| C8orf22 | 1,951943212 | 1,60E-05 |
| FSTL5 | -1,910904342 | 1,89E-07 |
| SOX14 | 2,092345498 | 3,74E-05 |
| ZBBX | 1,695793987 | 2,72E-05 |
| GP2 | 2,333735553 | 6,41E-07 |
| GJB1 | 1,578322975 | 2,37E-07 |
| FABP4 | -2,766383089 | 2,86E-17 |
| CST5 | 1,596474868 | 0,00024912 |
| DYDC1 | 1,80795456 | 1,14E-06 |
| MUC7 | -3,154186501 | 1,70E-11 |
| KRT20 | 1,664932697 | 5,98E-08 |
| WDR87 | 2,072795425 | 2,00E-09 |
| KRT2 | -1,508665605 | 1,61E-06 |
| INSM1 | 1,949822053 | 1,13E-13 |
| DPP10 | 2,248401809 | 1,77E-08 |
| DEFB103A | -1,773215354 | 0,00078207 |
| DEFB103B | -1,673292361 | 0,00035971 |
| FAM133A | -1,897671288 | 3,36E-06 |
| SAGE1 | -2,165431029 | 0,00013306 |
| PCP4 | 2,227798444 | 3,31E-08 |
| LCE3A | -2,17496985 | 5,26E-13 |
| LCE3E | -1,812773734 | 2,31E-10 |
| ZPBP2 | 2,224908098 | 3,62E-05 |
| TMEM212 | 1,913086056 | 5,96E-06 |
| LCE1A | -1,521552888 | 0,00022942 |
| LCE2A | -2,242960044 | 7,30E-09 |
| LCE2C | -1,782188021 | 9,13E-06 |
| LCE2D | -1,897256908 | 1,96E-06 |
| LCE3B | -1,933692459 | 0,00257275 |
| KRTAP9-8 | -2,192080256 | 0,00020905 |
| SLC18A3 | 1,869439887 | 3,35E-05 |
| C9orf152 | 1,515990417 | 1,09E-08 |
| MAGEB16 | -2,153085707 | 0,00361894 |
| PRB3 | 2,348610161 | 1,32E-24 |
| C1orf68 | -1,594594159 | 9,55E-09 |
| LOR | -2,025985928 | 1,94E-08 |
| KPRP | -2,080460787 | 2,16E-13 |
| CLPSL1 | 1,705209676 | 6,09E-05 |
| SPANXN5 | -1,594357112 | 0,00023011 |
| C9orf135 | 1,53622629 | 0,00046219 |
| KRTAP3-1 | 1,567960909 | 0,00010964 |
| TDRD15 | -2,153258852 | 0,00032531 |
| PSG3 | 1,587568754 | 6,20E-06 |
| CT45A5 | 1,959153513 | 0,00191955 |
| ANKRD66 | 1,592539646 | 4,88E-06 |
| LCE6A | -1,773676 | 3,81E-05 |
| KRTAP9-4 | -1,700963276 | 0,0006291 |
| KRTAP5-4 | -1,502901467 | 0,00047119 |
| LCE3C | -2,244637305 | 6,85E-06 |
| TMEM179 | 1,590151041 | 4,75E-08 |
| GFY | 1,740606044 | 7,30E-10 |
| MSMB | 1,944140214 | 1,01E-11 |
| FAM25C | -1,661719564 | 1,08E-07 |

Supplemental table 3: Differentially expressed genes of TCGA-HNSCC cohort according to angiolymphatic invasion status using limma package

| **Hugo Symbol** | **log2 Fold Change** | **FDR** |
| --- | --- | --- |
| SLC18A3 | 1,514351 | 0,00826763 |
| SPRR2G | -1,9168582 | 0,00832909 |
| SHH | 1,50486672 | 0,00826763 |
| PRR9 | -1,9473948 | 0,01047463 |
| SPRR2F | -1,7932964 | 0,01247376 |
| LCE3E | -1,6857211 | 0,01247376 |
| WFDC12 | -1,6759686 | 0,01391085 |
| LCE2B | -1,5935151 | 0,01404831 |
| SPRR2B | -1,7441298 | 0,01404831 |
| SPRR2E | -1,539815 | 0,01404831 |
| DSG1 | -1,5654636 | 0,01404831 |
| CLDN17 | -1,5232318 | 0,01576936 |
| LCE3D | -1,5340193 | 0,02107155 |
| OTOP3 | -1,5993729 | 0,02107155 |
| KRTDAP | -1,5472148 | 0,02240122 |
| DEFB4A | -1,633907 | 0,02796646 |
| KRT1 | -1,583375 | 0,0496268 |

Supplemental table 4: Chi squared test; HNSCC with available histological ALI status

| **Chi squared test, HNSCC with available histological ALI status** | | |
| --- | --- | --- |
| p-value = 2.451e-09 | Histological ALI negative | Histological ALI positive |
| Gene signature Cluster A | 151 | 42 |
| Gene signature Cluster B | 66 | 77 |

Supplemental table 5: Chi squared test; HNSCC subsites with available histological ALI status

| **Chi squared test, LaSCC with available histological ALI status** | | |
| --- | --- | --- |
| p-value = 0.0007729 | Histological ALI negative | Histological ALI positive |
| Gene signature Cluster A | 21 | 4 |
| Gene signature Cluster B | 20 | 30 |
| **Chi squared test, OSCC with available histological ALI status** | | |
| p-value = 0.0005085 | Histological ALI negative | Histological ALI positive |
| Gene signature Cluster A | 120 | 36 |
| Gene signature Cluster B | 37 | 33 |
| **Chi squared test, OPSCC with available histological ALI status** | | |
| p-value = 0.03282 | Histological ALI negative | Histological ALI positive |
| Gene signature Cluster A | 10 | 2 |
| Gene signature Cluster B | 9 | 14 |

Supplemental table 6: Chi squared test; Progression free survival of HNSCC according to gene expression signature

| **Chi squared test, HNSCC** | | |
| --- | --- | --- |
| p-value = 0.1636 | No recurrence | Disease progression |
| ALI Geneset Cluster A | 132 | 132 |
| ALI Geneset Cluster B | 101 | 132 |

Supplemental table 7: Chi squared test; Overall survival of HNSCC according to gene expression signature

| **Chi squared test, HNSCC** | | |
| --- | --- | --- |
| p-value = 0.0429 | Alive | Deceased |
| ALI Geneset Cluster A | 174 | 90 |
| ALI Geneset Cluster B | 132 | 101 |

Supplemental table 8: Chi squared test; Progression free survival of HNSCC subsites according to gene expression signature

| **Chi squared test, LaSCC** | | |
| --- | --- | --- |
| p-value = 0.004594 | No recurrence | Disease progression |
| Gene signature Cluster A | 25 | 11 |
| Gene signature Cluster B | 29 | 46 |
| **Chi squared test, OSCC** | | |
| p-value = 0.2221 | No recurrence | Disease progression |
| Gene signature Cluster A | 96 | 115 |
| Gene signature Cluster B | 35 | 59 |
| **Chi squared test, OPSCC** | | |
| p-value = 0.813 | No recurrence | Disease progression |
| Gene signature Cluster A | 11 | 6 |
| Gene signature Cluster B | 37 | 27 |

Supplemental table 9: Chi squared test; Overall survival of HNSCC subsites according to gene expression signature

| **Chi squared test, LaSCC** | | |
| --- | --- | --- |
| p-value = 0.01676 | Alive | Deceased |
| ALI Geneset Cluster A | 28 | 8 |
| ALI Geneset Cluster B | 39 | 36 |
| **Chi squared test, OSCC** | | |
| p-value = 0.06594 | Alive | Deceased |
| ALI Geneset Cluster A | 133 | 78 |
| ALI Geneset Cluster B | 48 | 46 |
| **Chi squared test, OPSCC** | | |
| p-value = 0.8431 | Alive | Deceased |
| ALI Geneset Cluster A | 13 | 4 |
| ALI Geneset Cluster B | 45 | 19 |

Supplemental table 10: Chi squared test; LaSCC with available histological ALI status

| **Chi squared test, LaSCC** | | |
| --- | --- | --- |
| p-value = 0.05644 | Histological ALI negative | Histological ALI positive |
| ALI Geneset Cluster A | 21 | 4 |
| ALI Geneset Cluster B | 20 | 30 |

Supplemental table 11: Clinical and pathological characteristics of patients with LaSCC out of HIPO-HNSCC (in-house cohort). PNI = Perineural invasion, ECS = Extracapsular spread, S =Surgery, SRT = Surgery and Radiotherapy, SRCT = Surgery and Radiochemotherapy.

| **Patient** | **Gender** | **Age** | **Tobacco** | **Alcohol** | **HPV-related** | **T Status** | **N Status** | **Grade** | **Therapy** | **Resection Margin** | **PNI** | **ALI** | **ECS** |
| --- | --- | --- | --- | --- | --- | --- | --- | --- | --- | --- | --- | --- | --- |
| HNC-18 | male | 62 | yes | no | no | 4a | 2b | 2 | SRCT | 0 | 0 | 0 | yes |
| HNC-21 | male | 54 | yes | yes | no | 4 | 0 | 2 | SRCT | 0 | 0 | 0 |  |
| HNC-23 | male | 66 | yes | yes | no | 3 | 0 | 2 | SRT | 0 | 0 | 0 |  |
| HNC-37 | male | 64 | yes | no | no | 3 | 2c | 2 | SRCT | 0 | 0 | 1 | yes |
| HNC-43 | male | 69 | yes | yes | no | 3 | 0 | 2 | S | 0 | 0 | 0 |  |
| HNC-47 | female | 59 | yes | yes | no | 4a | 2b | 2 | SRT | 1 | 0 | 0 | no |
| HNC-55 | female | 83 | yes | no | no | 3 | 0 | 2 | S | 0 | 0 | 0 |  |
| HNC-62 | male | 64 | yes | no | yes | 4a | 0 | 2 | SRT | 0 | 0 | 0 |  |
| HNC-64 | male | 61 | yes | no | no | 4a | 2b | 2 | S | 0 | 0 | 0 | no |
| HNC-65 | male | 62 | yes | no | no | 4a | 0 | 3 | SRT | 0 | 0 | 1 |  |
| HNC-68 | male | 49 | yes | yes | no | 3 | 1 | 2 | SRCT | 0 | 0 | 1 | yes |
| HNC-75 | male | 51 | yes | yes | no | 3 | 0 | 2 | S | 0 | 0 | 0 |  |
| HNC-76 | male | 56 | yes | no | no | 4a | 0 | 3 | SRT | 0 | 0 | 0 |  |
